# Human health risks associated with the consumption of groundwater in the Gaza Strip

**DOI:** 10.1016/j.heliyon.2023.e21989

**Published:** 2023-11-08

**Authors:** Basem Shomar, Joaquim Rovira

**Affiliations:** a Environmental Science Center, Qatar University, P.O. Box: 2713 Doha, Qatar; b Environmental Engineering Laboratory, Departament d'Enginyeria Química, Universitat Rovira i Virgili, Paisos Catalans Avenue 26, 43007 Tarragona, Catalonia, Spain; c Laboratory of Toxicology and Environmental Health, School of Medicine, Universitat Rovira i Virgili, Sant Llorenç 21, 43201 Reus, Catalonia, Spain; d Institut d’Investigació Sanitaria Pere Virgili (IISPV), 43204, Reus, Catalonia, Spain

**Keywords:** Human health risks, Groundwater contamination, Monte Carlo simulation, Gaza

## Abstract

Groundwater of the Gaza Strip, the main source of drinking water for the Gazans, is highly contaminated by several chemicals of natural and anthropogenic origins. The results of this study confirm the findings of several studies conducted over the past two decades. Over those two decades, the population of Gaza has doubled, resulting in heavy demand for the limited reserves of groundwater. After 20 years since the first comprehensive study, it was found that groundwater salinity increased by 30 %, due to seawater intrusion. On the other hand, nitrate (NO_3_) decreased by 30 %, due to expansion of the sewer network and decrease in the number and distribution of septic tanks. Salinity, chloride (Cl), NO_3_ and fluoride (F) distribution maps for the year 2022 are very similar to those of the year 2002. This indicates that sources and loads of such contaminants are still the same. Metals and metalloids are still within the permissible limits set by the World Health Organization (WHO). Strontium (Sr) only showed concentrations of 12 mg/L across the Gaza Strip, which calls for further investigations. Maximum concentrations of the NO_3_ and F were 365 and 2.6 mg/L, respectively. The results of probabilistic risk assessment using Monte Carlo simulation showed that NO_3_ and F consumption through drinking water were above the reference dose for 35 % and 5 % of the trials performed, respectively. Consequently, the hazard quotient (*HQ*) is larger than 1 for 35 % and 5 % of the exposure scenarios simulated for these ions. For all metals and metalloids analyzed, *HQ* were below one (*HQ*1) indicating no risk; however, Sr presented an *HQ* 95th percentile equal to 0.19. Exposure routes such as dietary intake and soil ingestion, among others, should be further investigated to ensure that cumulative exposure does not surpass the safety limit. Recent advances in desalination technology should put an end to this truly regrettable situation.

## 1 Introduction

In the Mediterranean basin, climate change will lead to a decrease in precipitation [1]. This, together with anthropogenic influences, will result in water scarcity and salinization of the costal aquifers.

By the end of 2018, several international reports were issued, projecting that the Gaza Strip would not be habitable within two years due to the absence of freshwater resources [2]. Over the past 25 years, several water-related research projects have been implemented in the Gaza Strip. The area faces enormous long-standing and emerging challenges resulting from a very high population density, contaminated groundwater, lack of appropriate wastewater treatment and disposal sites, and lack of reliable electricity and chemical supplies necessary to implement modern water purification technologies, among other issues. Access to clean water is among the bare minimum requirements to maintain an acceptable quality of life. On March 22, 2022, the Palestinian Central Bureau of Statistics and the Palestinian Water Authority issued a joint press release on the occasion of the UN World Water Day, which came under the slogan “Groundwater-Making the Invisible Visible” [3]. The statement revealed recent findings on water budget and quality in the West Bank and the Gaza Strip.

The estimated amount of extracted groundwater in 2020 in the Gaza Strip was 190.5 million m^3^, of which more than 97 % did not meet the WHO standards [4]. This corresponds to 86.6 L per capita per day (L/c/d), of which only 26.8 was fresh water [5]. To respond to the increasing demand for fresh water, Gaza established multiple new desalination plants [6,7]. In 2020, Gaza produced 5.7 million m^3^ of desalinated water using seawater reverse osmosis (SWRO) technology [8]. More desalination plants will produce more water in the coming years [3].

Numerous studies have been carried out in the past to assess water quality in the Gaza Strip, which has one of the highest population densities in the world (5320 people/km^2^) and with highly depleted and deteriorating groundwater resources [9,10].

The health aspects of drinking water are very demanding in Gaza, and the call for “Sanitation for all” by Narain [11] supports such dimensions. The health situation of the Palestinians was addressed in a series of important articles [[12], [13], [14], [15]]. Though highly pertinent, these articles directly link water issues to public health in Gaza.

More than one-third of the population of Gaza is less than 15 years old [15] with the majority being school kids. Recently, the United Nations reported that drinking water is not available to all school kids. One out of every three schools cannot provide the basic needs of fresh drinking water and sanitary facilities for washing hands with soap and water [16,17].

The scarcity of land allows wastewater to drain among residential areas or on open land forming lakes, poisoning farms and the food chain threatening to kill the surrounding population [18].

NO_3_ intake through drinking water and their capacity to be reduced into nitrite, has the capacity to promote formation of methemoglobin with a decreased capacity to transport oxygen especially in infants [19]. In addition, NO_3_ in drinking water has been reported to be linked to risks of type I diabetes, endocrine and developmental effects, and even cancer [19]. Furthermore, long-term consumption of F in drinking water may lead to the development of dental and skeletal fluorosis [9]. Other studies showed that F concentrations in drinking water could be associated with the development of some cancers [20].

Despite the seriousness of the water situation in Gaza, and the large number of available studies and international reports, there are no adequate in-depth studies on the human health risks of contaminated drinking groundwater. Efforts of national and international experts, researchers and scientists focus on specific contaminants, including microorganisms [12], NO_3_ [16], and F [8].

Consequently, the novelty of this study stems from the geographical location it focuses on. The geopolitical situation is unique and understanding and mitigation of the water crisis is very challenging to governments and UN entities. Although the study avoided politics, all UN reports confirmed that the cornerstone for solving the water problems (and others) is solving the political conflict in the region [4]. Additionally, the novelty lies in the interlinkages between the absence of fresh water, electric power shortages, the area having the highest population density in the world and the basic needs of food in a very limited and closed area, etc.

Recent studies discussed the importance of health risk elements of groundwater contaminants. It has been found that heavy metals are contributing to the non-carcinogenic risks in people consuming groundwater [21]. Risks of toxic elements such as As, Cd, Cr, Cu, Fe, Mg, and Mn in drinking groundwater were investigated by Eslami et al. [22]. The study found that the non-carcinogenic risk of As in drinking groundwater is high and, generally, As is the major risk factor to water consumers. During the same year, in the district Hyderabad (Pakistan) hazard quotient (*HQ*) was used for assessing the risks of Cu, Ni, As, Pb, Cd and Zn in groundwater. The results showed that non-carcinogenic risks were below the recommended *HQ* threshold [23]. In India, the risks of elements Fe, Mn, Zn, B, As, Ni, and Pb in groundwater of urban Delhi were investigated. Surprisingly, the hazard index values for the tested elements were found to be significantly high [24]. In the Haridwar district, the study of Khan and Rai [25] found that 25 % of groundwater is associated with non-carcinogenic health risks caused predominantly by As, Fe, Pb, and Cd contamination, while all samples had carcinogenic health risks due to As, Cd, and Cr. Additionally, Soleimani et al. [26] focused on the human health risks of NO_3_ in groundwater and found that it is the critical non-carcinogenic risk for all people exposed to it.

Therefore, this study comes to expose a wide spectrum of chemical pollutants in the groundwater of the Gaza Strip, being the main source of drinking for the local inhabitants, and the health risks associated with these contaminants. It also, for the first time in Gaza, combines field and laboratory findings with simulation models to clearly highlight the significance of the issues of water in Gaza and the region in general.

Consequently, the main objectives of the study include: (i) an updated chemical characterization of the groundwater in the Gaza Strip compared to the one in 2002, and (ii) calculation of the human exposure to potentially toxic ions and elements, and their associated health risks through consumption of drinking water, in a probabilistic way using Monte Carlo simulations.

## 2 Methodology

### 2.1 Sampling campaigns and field measurements

Based on the local conditions of the Gaza Strip, the authors proposed a scientific and achievable method to conduct the work. The method includes collection of groundwater samples from the targeted 115 wells and analysis of samples in the laboratories using quality control/quality assurance (QA/QC) analytical protocols.

**Fig. 1:**
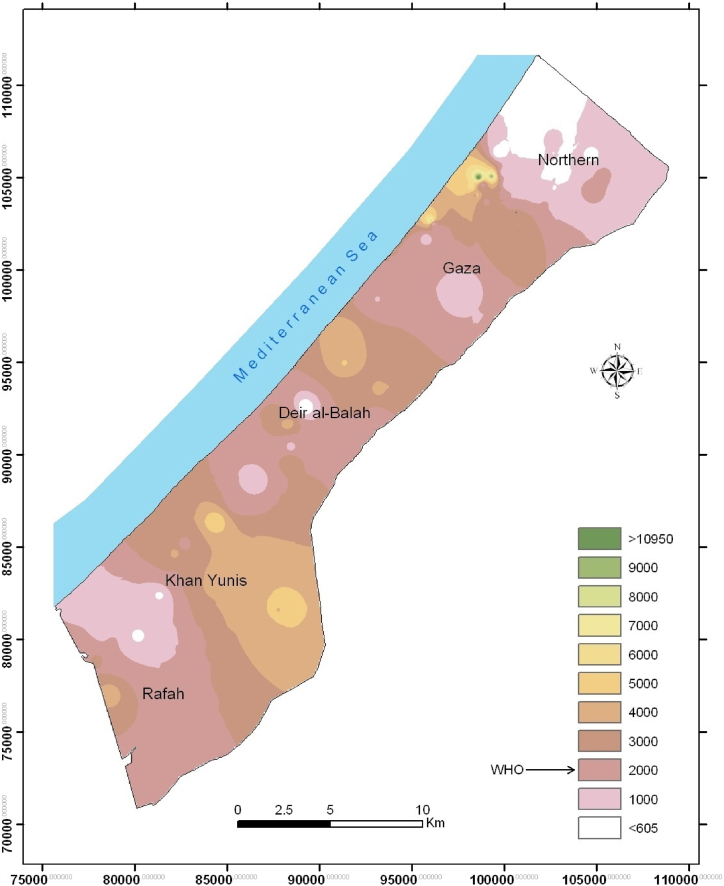
Spatial distribution of EC (*μ*S/Cm) in the groundwater of the Gaza Strip (2022).

In full coordination with the Palestinian Water Authority (PWA) and lately the Coastal Municipalities Water Utility (CMWU) Gaza, 115 municipal groundwater wells were selected in October 2022. The Gaza Strip has five governorates (Fig. 1). The selected wells are distributed as follows: 30 in the Northern Governorate, 34 in Gaza Governorate, 18 in Deir al-Balah Governorate, 22 in Khan Yunis Governorate and 11 in Rafah Governorate. The history of the selected wells such as the name of the owner, the geographical coordinates, the year of digging, and the utilization rates have been recorded. The selected wells are routinely monitored by PWA and CMWU for basic parameters such as salinity, total dissolved solids (TDS), pH, EC, Cl and NO_3_ [9,10,27]. Prior to the sampling process, each groundwater well was operated for 15–20 min to rinse pipes and assure continuous pumping from the groundwater itself and not from the suspended water in the pipes. Water samples were collected in acid-washed plastic bottles for cations and metals while a second set of sterilized bottles were used for the determination of anions. Each sampling container was washed three times with the groundwater of the well before filling and storing in a sampling icebox. Physicochemical parameters, including pH, EC and TDS of each sample were determined using portable probes and results were taken [9].

### 2.2 Laboratory work and sample analyses

Upon arrival to the labs of the CMWU, Cl and NO_3_ were determined by titration methods [28]. Same samples were shipped to the University of Heidelberg, Germany where F, Br, PO_4_ and SO_4_ were determined by an ion chromatograph (IC) (Dionex ICS-1100; Thermo Scientific Dionex, USA). Metals and metalloids were determined by an inductively coupled plasma mass spectrometry ICP-MS (Agilent 7500ce and 7700x; Agilent Technologies, Inc., USA). Quality control (QC) and quality assurance (QA) protocols were implemented [29,30]. Table 1 shows the list of parameters and basic statistical results.

**Table 1:** Groundwater quality in the Gaza Strip (n = 115).

	Unit	Max.	Min.	Mean	Median	Standard deviation	WHO [38]
Physical Indicators							
pH		8.08	6.65	7.45	7.46	0.30	6.5–9.5
EC	*μ*S/Cm	29600	709	5202	3750	5101	2000
TDS	mg/L	18352	354.5	3261	2325	3187	1000
Hardness	mgCaCO_3_/L	2434	82	467	398	322	200
***Major Anions***							
Cl	mg/L	9491	91	1419	810	1797	250
NO_3_	mg/L	365	16	112	93	74	50
F	mg/L	2.6	0.2	1.1	1.1	0.53	1.5
Br	mg/L	76.5	0.03	3.54	0.25	12.7	62
PO_4_	mg/L	72	72	72	72	a	–
SO_4_	mg/L	1537	7.8	177	119	199	250
HCO_3_	mg/L	557	132	294	275	87	–
***Major Cations***							
Ca	mg/L	375	13	84	68.4	60	75
Mg	mg/L	400	10.7	58	46.8	46	50
Na	mg/L	430	22.3	180	176	101	50
K	mg/L	48	1.17	7.46	4.82	7.72	12
***Metals and Metalloids***							
Ag	*μ*g/L	2.1	1.7	1.9	1.9	0.13	100
Al	*μ*g/L	<0.02	<0.02	<0.02	<0.02	<0.02	200
As	*μ*g/L	<16	<16	<16	<16	<16	10
Ba	*μ*g/L	417	17.9	146	143	90	1300
Cd	*μ*g/L	<0.3	<0.3	<0.3	<0.3	<0.3	3
Co	*μ*g/L	<1.7	<1.7	<1.7	<1.7	<1.7	20
Cr	*μ*g/L	102	6.9	26	21.3	16.7	50
Cu	*μ*g/L	18	18	18	18	a	2000
Fe	*μ*g/L	430	0.13	68	87	57	300
Mn	*μ*g/L	35.8	0.06	1.56	1.28	3.33	0.8
Ni	*μ*g/L	<15	<15	<15	<15	<15	70
Pb	*μ*g/L	<3	<3	<3	<3	<3	10
Sr	*μ*g/L	12350	539	2521	1770	2049	–
Zn	*μ*g/L	61.2	1.42	9.7	7.59	8.51	3000

a Only 1 sample showed detectable concentrations for PO_4_ and Cu.

### 2.3 Human health risk assessment

Exposure to chemicals (*i*) through drinking water (*Exp*_*drink,i*_) was calculated using Eq. (1) [30].

(1) $${\mathrm{Exp}}_{drink,i}=\frac{C_{i}\times I_{w}}{BW}$$

where *C*_*i*_ is the concentration level of chemical *i*, *I*_*w*_ is the water intake and *BW* is the body weight.

To deal with uncertainty and statistical distribution of the data, the exposure was calculated in a probabilistic way using Monte Carlo simulation. This approach was recently successfully applied for human health risk assessment [[31], [32], [33]]. Using the results of each parameter, a logarithmic normal distribution was used to calculate risks. For adult male and female, a daily water consumption between 0.5 and 2 L was considered [30]. For children of a 1–3 years old, a daily water consumption of 0.25 and 1.0 L was considered. Finally, for adult male and adult female body weights, a logarithmic normal distribution with a median of 80 Kg with 95th percentile of 100 Kg and median 65 Kg with 95th percentile of 85 Kg was assumed. Consequently, a median value of 12.5 Kg with a 95th percentile of 20 Kg was considered for children between 1 and 3 years old.

To perform the Monte Carlo simulation, Oracle Crystal Ball© software (version 11.1.2.4.850) was employed. For each calculation, 100,000 iterations were performed.

To assess systemic risk, hazard quotients (*HQ*_*i*_) were calculated using Eq. (2).

(2) $${HQ}_{i}=\frac{{\mathrm{Exp}}_{drink,i}}{{RfD}_{o,i}}$$

Where *RfD*_*o,i*_ is the most updated oral reference dose for a given element established by USEPA [34]. A value of *HQ*_*i*_ below 1 indicates a non-risky exposure for a given chemical *i*.

The carcinogenic risks were not calculated due to the fact that all potentially carcinogenic elements were very low and all were below the instrumental detection limit. Moreover, only total chromium (Cr) was analyzed and no information were obtained for the hexavalent chromium contents.

## 3 Results and discussion

### 3.1 General readings of physical indicators

Results of general physicochemical parameters for all wells are given in Supplementary Table S1. Table 1 summarizes the key statistical findings of the well water quality in the Gaza Strip. Generally, groundwater of the Gaza Strip has values of total dissolved solids (TDS), electrical conductivity (EC) and hardness above the levels permissible by the WHO guidelines [35]. Only a few wells in the northern parts of Gaza are found to meet the acceptable concentrations by the WHO (Table 1 and Fig. 1). Such results confirm the general impression of public community and policy makers about the brackish nature of groundwater in Gaza [36,37]. The water is salty (mean TDS is 3261 mg/L) and hard (mean hardness is 467 mgCaCO_3_/L) which makes it unsuitable for human consumption as drinking water and even unfit for basic daily domestic purposes [38].

Several modeling and simulation tools have been used to investigate the sources of salinity in the groundwater of Gaza. It is well documented that high salinity concentrations in the groundwater of the Gaza Strip is mainly due to seawater intrusion and saltwater up-coming [39,40] coupled with the over exploitation of such limited resource [41]. The study of Ghabayen et al. [42] used Na/Cl, SO_4_/Cl, Br/Cl, Ca/(HCO_3_+SO_4_), and Mg/Ca ionic ratios to distinguish different sources of salinity. The study used models for δ^11^B and ^87^Sr/^86^Sr isotopic composition. All studies concluded that groundwater salinity would increase over time due to seawater intrusion. Comparing the results of 2002 with the results of 2022 for groundwater salinity (represented by TDS) of the same wells show that salinity increased by 31 % over the 20 years. As the population in Gaza has doubled in the past two decades, more groundwater has been overexploited and more seawater invaded the aquifers and increasing salinity.

### 3.2 Groundwater anions, cations, metals and metalloids

**Fig. 2:**
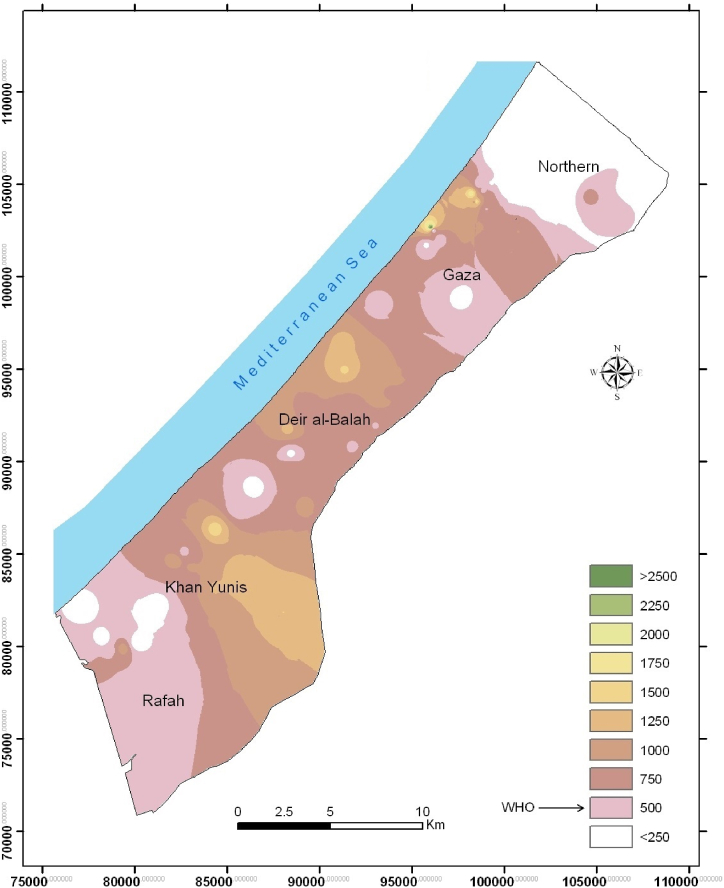
Spatial distribution of Cl (mg/L) in the groundwater of the Gaza Strip (2022).

**Fig. 3:**
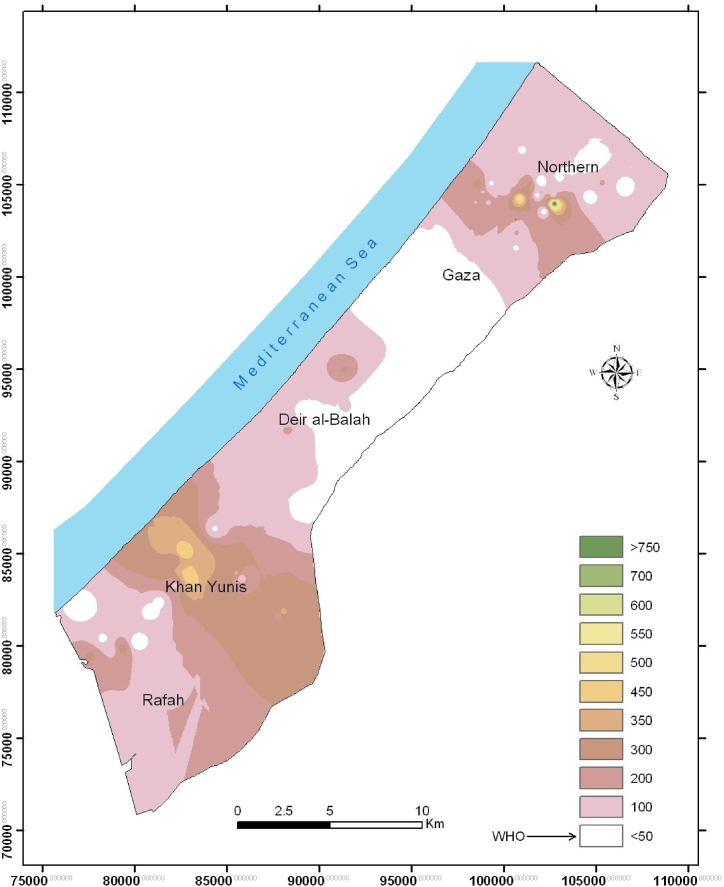
Spatial distribution of NO_3_ (mg/L) in the groundwater of the Gaza Strip (2022).

It is well known that increasing trend of major ions is associated with increasing trend of groundwater salinity. As shown in Table 1, the mean concentrations of Cl (Fig. 2) and NO_3_ (Fig. 3) for all sampled wells are higher than the permissible limits set by the WHO, revealing that groundwater in the Gaza Strip is not suitable for human consumption as drinking water. Adding the results of EC and TDS to the results of Cl (1419 mg/L), NO_3_ (112 mg/L) and one well PO_4_ (72 mg/L) revealed that such groundwater is not drinkable. For the well contaminated with PO_4_ in 2002 and 2022, it is believed that a pollution point source could be the reason. This may include storing phosphate fertilizers in the well territory or leaching of PO_4_ to the groundwater from the surrounding regions. Results of anions, cations, metals and metalloids are given in supplementary tables S1, S2 and S3.

It is worth mentioning here that having only one analyte or two below the WHO guidelines is not a true representation of the universal water contamination problem in Gaza. This is clear with the mean concentrations of Br (3.5 mg/L) and SO_4_ (177 mg/L), where some wells have acceptable ranges but the concentrations of other analytes are found to be much higher than the values recommended by the WHO.

The concentrations of both Cl and Na are high (Table 1) and are attributed to seawater intrusion [42]. In a previous study, atrazine was detected in a few wells of the Gaza Strip [43] and, furthermore, a linear correlation between Cl concentrations and those of atrazine in the same wells was found.

Public health studies confirmed the presence of a few diseases which may be attributable to drinking water contamination by chemicals in Gaza. The first example is related to the NO_3_ concentrations in drinking water and the presence of methemoglobinemia (MetHb) among infants who drink milk formulae with NO_3_ coming from groundwater (Fig. 3). According to Shomar et al. [17], NO_3_ sources in the groundwater of Gaza originated mainly from manure released from septic tanks and/or from synthetic fertilizers stored or applied in nearby farms. NO_3_ mean concentrations for the same wells decreased by 30 % in the past 20 years. This can be explained by the extension of sewage collection network and the decrease in agricultural activities due to urbanization and population growth.

**Fig. 4:**
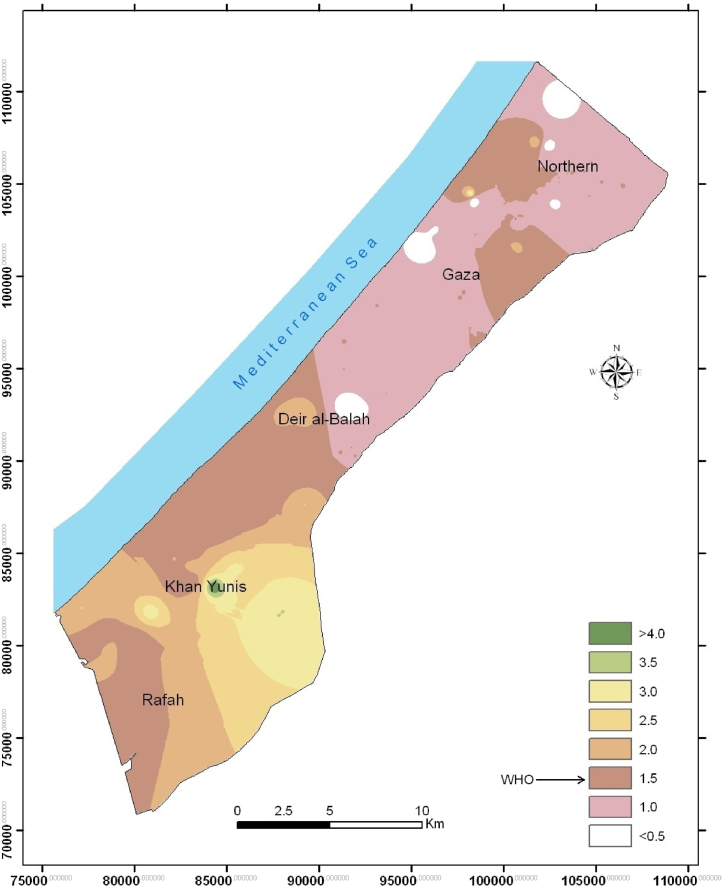
Spatial distribution of F (mg/L) in the groundwater of the Gaza Strip (2022).

The second example is the dental fluorosis among school kids in the southern parts of the Gaza Strip, due to the high concentrations of F anion in drinking water (Fig. 4). It has been found that high F concentrations in the groundwater especially in the southern parts of the Gaza Strip and were attributed to the presence of naturally available calcium fluoride (CaF_2_) [9].

All tested metals and metalloids in the groundwater of the Gaza Strip are below the instrumental detection limit (Table 1) or within the permissible limits of the WHO [35]. In a few cases, for some wells, some metals were found in concentrations that exceed the limits recommended by WHO. For example, chromium was detected in concentrations up to102 μg/L which are 2 times of the WHO recommended limit (50 μg/L). Likewise, the concentrations of Fe and Mn were found to be 430 μg/L and 36 μg/L, respectively, higher than the lower limits recommended by WHO (Table 1). Although samples were collected over four times, Cu was detected in one well only with mean concentrations of *μ*g/L, which is much lower than the permissible limit of the WHO (2000 μg/L). On the other hand, the WHO has no guidelines for Sr in drinking water, while the Agency for Toxic Substances and Disease Registry (ATSDR) [44] recommended that Sr should not exceed 4 mg/L. Several wells scattered in Gaza showed mean concentrations of 12 mg/L. Such results call for further investigations on Sr sources and potential health risks. Few recent studies found that Sr is naturally occurring in groundwater and the high concentrations of Sr in groundwater of the USA and China is due to water-rock interaction, as well as saline groundwater mixing [45,46].

### 3.3 Exposure and human health risk assessment

Probabilistic exposures to anions, cations and chemical elements are summarized in Table 2. Exposure assessment was only performed for the elements that were detected in significant concentrations in the groundwater samples. Ions of Cl, Na, SO_4_, NO_3_ and Ca showed the highest median values while Sr showed the highest median intake levels for metals (0.026; P95: 0.200 mg/kg/day).

**Table 2:** Probabilistic adult male exposure (mg/kg/day) to ions and elements through drinking water and oral reference dose (mg/kg/day).

Parameter	Mean	SD	P50	P75	P90	P95	P99	RfDo [34]
**Cl**	2.7x10^1^	5.7x10^1^	1.2x10^1^	2.8x10^1^	6.1x10^1^	9.6x10^1^	2.3x10^2^	
**NO**_**3**_	1.8x10^0^	1.4x10^0^	1.4x10^0^	2.3x10^0^	3.5x10^0^	4.5x10^0^	7.1x10^0^	1.6x10^0^
**F**	1.9x10^−2^	1.2x10^−2^	1.6x10^−2^	2.4x10^−2^	3.4x10^−2^	4.1x10^−2^	5.9x10^−2^	4.0x10^−2^
**Br**	1.5x10^−2^	2.2x10^−1^	3.6x10^−4^	2.3x10^−3^	1.2x10^−2^	3.3x10^−2^	2.2x10^−1^	
**PO**_**4**_	4.0x10^−4^	1.6x10 ^−4^	3.9x10 ^−4^	5.1x10 ^−4^	6.1x10 ^−4^	6.7x10 ^−4^	7.8x10 ^−4^	
**SO**_**4**_	2.7x10^0^	3.2x10^0^	1.8x10^0^	3.3x10^0^	5.8x10^0^	8.3x10^0^	1.6x10^1^	
**Ca**	1.3x10^0^	1.0x10^0^	1.0x10^0^	1.7x10^0^	2.5x10^0^	3.2x10^0^	5.1x10^0^	
**Mg**	8.8x10^−1^	6.8x10^−1^	7.0x10^−1^	1.1x10^0^	1.7x10^0^	2.2x10^0^	3.4x10^0^	
**Na**	3.0x10^0^	1.8x10^0^	2.6x10^0^	3.9x10^0^	5.3x10^0^	6.4x10^0^	8.9x10^0^	
**K**	1.2x10^−1^	1.8x10^−1^	7.1x10^−2^	1.5x10^−1^	2.7x10^−1^	4.0x10^−1^	8.4x10^−1^	
**Ag**	1.4x10^−5^	5.7x10^−6^	1.3x10^−5^	1.7x10^−5^	2.1x10^−5^	2.4x10^−5^	2.9x10^−5^	5.0x10^3^
**Ba**	2.6x10^−3^	1.8x10^−3^	2.1x10^−3^	3.3x10^−3^	4.8x10^−3^	6.0x10^−3^	9.0x10^−3^	2.0x10^−1^
**Cr**	4.0x10^−4^	3.1x10^−4^	3.2x10 ^−4^	5.1x10 ^−4^	7.7x10^−4^	9.9x10^−4^	1.6x10^−3^	1.5x10^0^
**Fe**	1.4x10^−3^	6.2x10^−4^	1.3x10^−3^	1.8x10^−3^	2.3x10^−3^	2.6x10^−3^	3.2x10^−3^	7.0x10^−1^
**Mn**	2.2x10^−5^	1.4x10^−5^	1.9x10^−5^	2.9x10^−5^	4.0x10^−5^	4.9x10^−5^	7.0x10^−5^	2.4x10^−2^
**Sr**	3.8x10^−2^	4.1x10^−2^	2.6x10^−2^	4.7x10^−2^	8.1x10^−2^	1.1x10^−1^	2.0x10^−1^	6.0x10^−1^
**Zn**	1.4x10^−4^	1.1x10^−4^	1.1x10^−4^	1.8x10^−4^	2.8x10^−4^	3.6x10^−4^	5.6x10^−4^	3.0x10^−1^
SD: Standard deviation; P50, P75, P90, P95, P99: Percentile 50th, 75th, 90th, 95th, 99th; RfDo: Reference oral dose.								

**Fig. 5:**
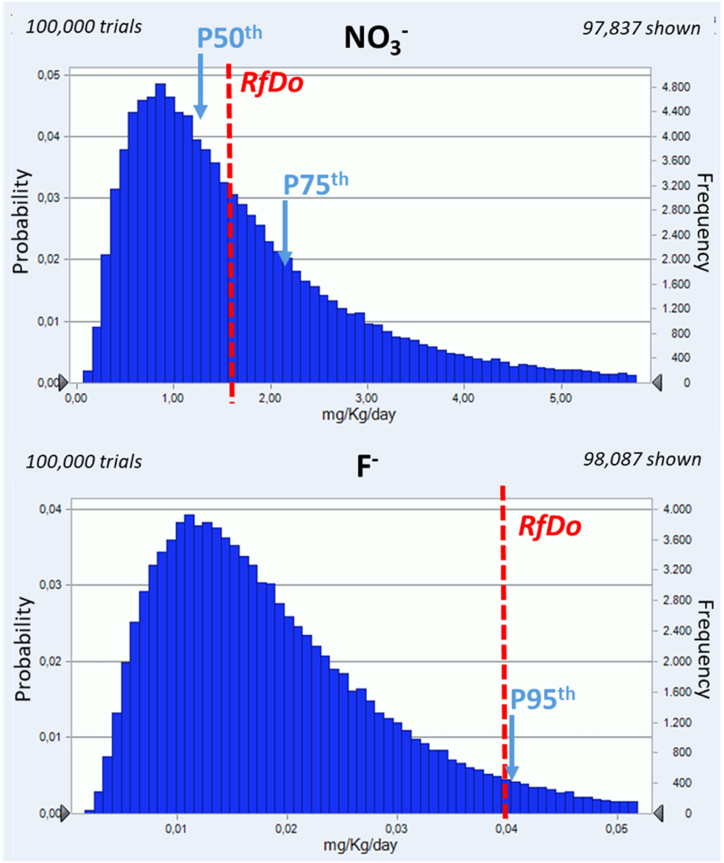
Adult male exposure distribution of NO_3_ (up) and F (down) ions with respective to oral reference dose (RfDo) for these ions set by the USEPA [34] in red. (For interpretation of the references to colour in this figure legend, the reader is referred to the Web version of this article.)

Regarding the non-carcinogenic risks, all *HQ*_*i*_ were far below 1, less than 0.05 in the 95th percentile of the exposure, for all elements and ions except for NO_3_ and F. For both ions, their exposure exceeds their corresponding oral reference dose established by the United States Environmental Protection Agency (USEPA) [34] (Fig. 5). In Fig. 5, the probabilistic distribution of the 100,000 trials performed for NO_3_ and F intake (in mg/kg/day) through drinking water consumption, using Monte Carlo simulation, are presented. The horizontal axis shows the exposure to a given element/ion through drinking water consumption (in mg/kg/day) while the vertical axis shows the probability or frequency of the exposure). At least a 65th percentile of NO_3_ exposure trials exceed the reference dose (1.60 mg/kg/day) marked in red. This means that in 35 % of the tests performed the amount of NO_3_ ingested were above the oral reference dose established by USEPA (1.60 mg/kg/day). However, the World Health Organization (WHO) and the European Food Safety Authority (EFSA) established an acceptable daily intake of 3.7 mg/kg/day [47,48]. Considering the reference value set by the WHO and the EFSA, around 10 % of the cases calculated in the present simulation still exceed this limit. Consequently, exposure to the NO_3_ should be considered unsafe. Similarly, 95th percentile of F exposure through drinking water surpasses the reference dose set by USEPA at 0.04 mg/kg/day (Fig. 5). EFSA established F adequate intake in 0.05 mg/kg/day for children and adults. However, this value covers all exposure sources such as diet, toothpaste, and other dental care products [49]. Despite the fact that Sr intake through drinking water does not exceed the oral reference dose for the 90th, 95th and 99th percentile of trials performed using probabilistic calculations, *HQ* for Sr were 0.13, 0.19 and 0.33, respectively. This indicates that for only one exposure route (drinking water) up to 33 % of the Sr oral reference dose was reached. Risk assessment should be performed considering other exposure routes such as dietary intake or soil and dust ingestion.

Considering other population groups, exposure to these elements for adult women increases the exposition of ions and elements up to 18.7 % due to the same water consumption and lower body weight (65 kg). However, considering children between 1 and 3 years old, the increase in the exposition was around 220 %. Despite children's water intake (0.25–1 L) being less than an adult, the lower body weight (12.5 kg) lead to an exposure through drinking water much higher than an adult. Special attention should be paid to children as a vulnerable population group and the high exposure assessed.

Finally, the study found that the number of municipal groundwater wells used for drinking purposes in Gaza have been increased to respond to the population growth and increasing water demand. The changes in the main parameters of groundwater quality in Gaza over the past 20 years are minimum. However, salinity increased by 30 % and NO_3_ mean concentrations decreased by 30 %. The study introduced for the first time computational tools (Monte Carlo simulation) to estimate the health risks associated with the consumption of drinking water in Gaza. Such tools are new to the region and outcomes based on them can be very valuable to the public, scientists and decision makers.

Table 3 summarizes the hazard quotients (*HQ*) distribution of the three population groups. In women and children, *HQ* regarding the exposure through drinking water of NO_3_ and F are exceeding the safety threshold (*HQ* = 1) for more than 50 % of the simulations performed.

**Table 3:** Probabilistic distribution of hazardous quotients (*HQ*) due to exposure od ions and elements through drinking water consumption in the Gaza Strip.

	P25	P50	P75	P90	P95	P99
Adult male						
NO_3_	0.53	0.86	1.41	2.17	2.80	4.44
F	0.27	0.41	0.61	0.85	1.03	1.47
Ag	<0.01	<0.01	<0.01	<0.01	<0.01	0.01
Ba	0.01	0.01	0.02	0.02	0.03	0.05
Cr	<0.01	<0.01	<0.01	<0.01	<0.01	<0.01
Fe	<0.01	<0.01	<0.01	<0.01	<0.01	<0.01
Mn	<0.01	<0.01	<0.01	<0.01	<0.01	<0.01
Sr	0.02	0.04	0.08	0.13	0.19	0.33
Zn	<0.01	<0.01	<0.01	<0.01	<0.01	<0.01
**Adult female**						
NO_3_	0.65	1.06	1.74	2.67	3.44	5.46
F	0.34	0.51	0.75	1.05	1.27	1.81
Ag	<0.01	<0.01	<0.01	0.01	0.01	0.01
Ba	0.01	0.01	0.02	0.03	0.04	0.06
Cr	<0.01	<0.01	<0.01	<0.01	<0.01	<0.01
Fe	<0.01	<0.01	<0.01	<0.01	<0.01	0.01
Mn	<0.01	<0.01	<0.01	<0.01	<0.01	<0.01
Sr	0.03	0.05	0.10	0.17	0.23	0.41
Zn	<0.01	<0.01	<0.01	<0.01	<0.01	<0.01
**Children from 1 to 3 years old**						
NO_3_	1.68	2.77	4.52	6.95	8.95	14.2
F	0.87	1.32	1.96	2.73	3.31	4.71
Ag	0.01	0.01	0.01	0.01	0.02	0.02
Ba	0.02	0.03	0.05	0.08	0.10	0.14
Cr	<0.01	<0.01	<0.01	<0.01	<0.01	<0.01
Fe	<0.01	0.01	0.01	0.01	0.01	0.01
Mn	<0.01	<0.01	<0.01	0.01	0.01	0.01
Sr	0.08	0.14	0.25	0.43	0.59	1.05
Zn	<0.01	<0.01	<0.01	<0.01	<0.01	0.01
P25, P50, P75, P90, P95, P99: Percentile 25th^,^ 50th, 75th, 90th, 95th, 99th; RfDo: Reference oral dose.						

### 3.4 Future of water in the Gaza Strip: strategic initiatives for water security

In the past few years, Gaza entered the era of seawater desalination technology. The SWRO desalination plants put an end to a truly regrettable situation and opened wide horizons for an improved quality of life in the region. In just four years, SWRO desalination plants have spread throughout the Gaza Strip, both large ones run by official authorities and smaller ones owned and operated by private companies. By mid- 2022, more than 35 % of the population of the Gaza Strip had desalinated water for drinking and household purposes. Currently, there are 154 brackish groundwater desalination plants, in addition to three seawater plants, with a combined production capacity of 22,000 m^3^/day. The desalination plants must increase at the pace that meets the population's ever-growing demand. Environmental and geopolitical risks will remain and may increase, but the presence of fresh water that meets the needs of the population is a great responsibility that must take precedence over most other tasks.

The observations, technical discussion and experience highlight the needs to address a few aspects to assure sustainable development of the water sector in Gaza mainly employing the desalination technology. Groundwater should stay as the strategic water reserve for the people of Gaza and should be protected, monitored, and regulated by public water entities and governmental institutions. The challenges of water security in Gaza are intertwined. All of them are real, given that the overall situation and political stability are very fragile. Selected challenges related to desalination plants that need special care and long-term investment can be summarized as follows: (1) desalination plants and political stability; (2) operation and maintenance; (3) environmental and health impacts; (4) international and regional cooperation and; (5) human resources capacity building.

Finally, it is important to mention the strengths and limitations of the study which may open horizons for further work. The strengths include comprehensive monitoring of groundwater in one of the most politically-volatile regions in the world, following major social and geopolitical changes spanning 20 years. Strategic and international partnerships can bridge the gap between the available resources (human and infrastructure) in Gaza and the state-of-the-art laboratories in Germany. The introduction of a new concept merging field/laboratory work with computation/simulation tools is new to the region. The concept addresses scientific messages and recommendations with minimum cost. Assessment of risks associated with water contamination and environmental deterioration using such tools in poor countries is very critical to help people and decision makers. Highlighting the water challenges in Gaza at the global level may call for support to the people directly or indirectly (e.g., building new desalination plants). On the other hand, the study points to the absence of a strategic vision related to water security, the weakness of monitoring programs, the absence of qualified laboratories where all needed work can be done locally, the absence of budget for scientific research to target more problems and find scientific solutions.

## 4 Conclusions

The groundwater in the Gaza Strip contains various chemical contaminants. However, the presence of some of these contaminants, even if they fall within WHO standards, does not necessarily ensure that the water is suitable for drinking. The treatment technology needed to convert such water to be drinkable and all high concentrations of contaminants should be targeted to meet the drinking water guidelines.

In 20 years, the mean salinity of groundwater increased by 30 % due to overexploitation and seawater intrusion while mean NO_3_ concentrations decreased by 30 % due to expansion in the sewer system and decrease in the number of septic tanks.

Probabilistic exposure assessment confirmed that intake of NO_3_ and F through drinking water were above the reference dose and, consequently, *HQ*_*i*_ could be larger than 1 in 35 % and 5 % of the cases considered in our statistical simulations. No risk was detected for any metal. However, Sr showed a percentile 95th value of 0.19. Exposure assessment should consider other pathways such as dietary or soil and dust ingestion to establish a complete exposure assessment of these chemicals. Exposure to NO_3_, F and metals such as strontium (Sr) should be further studied in the region and their concentrations reduced in drinking water to protect the health of the human population. Vulnerable population, such as young children, presented higher exposition due to higher water intake: body weight ratio and in consequence more potentially impacted.

In just four years, SWRO desalination plants have spread throughout the Gaza Strip and water security began to recover through international grants that contributed to the construction of such plants. Clear strategies should be adopted and practical steps ought to be takes to ensure the operation of desalination plants, in light of the complex conditions in the Gaza Strip, whether political, financial or operational. These strategies should be extended to cover the health, social and environmental aspects associated with desalination technology.

The study covers a broad spectrum of chemical parameters and concludes that an affordable and achievable roadmap is needed to protect water and human health. The studies of 2002, 2022 and all available reports are essential to identify the gaps, highlight the research questions and propose action plans.

Several hurdles still remain, such as the absence of strategic visions dealing with water challenges in Gaza in terms of quality and quantity. Qualified laboratories to conduct the work in a national monitoring program is absent, and the need for more qualified individuals who can merge field/lab work with theoretical tools, is clear.

## Ethical approval

This article does not contain any studies with human or animals performed by any of the authors.

## Funding

No external funding support has been received for this specific article.

## Data availability statement

Data included in article/supp. material/referenced in article.

## CRediT authorship contribution statement

**Basem Shomar:** Writing – review & editing, Writing – original draft, Supervision, Methodology, Formal analysis, Data curation, Conceptualization. **Joaquim Rovira:** Writing – review & editing, Writing – original draft, Visualization, Validation, Software, Data curation.

## Declaration of competing interest

The authors declare that they have no known competing financial interests or personal relationships that could have appeared to influence the work reported in this paper.
