# Supplementary material for: Human health risks associated with the consumption of groundwater in the Gaza Strip
Source: Heliyon. 2023 Nov 8;9(11):e21989. doi: 10.1016/j.heliyon.2023.e21989 (PMC10682630; doi:10.1016/j.heliyon.2023.e21989)
Supplement: Multimedia component 1 [file mmc1.docx]

Supplementary Material

**S1:** Results of Physical Parameters and Major Anions

|  | **pH** | **EC** | **TDS** | **HCO_3_** | **Hardness** | **Cl** | **NO_3_** | **F** | **Br** | **PO_4_** | **SO_4_** |
| --- | --- | --- | --- | --- | --- | --- | --- | --- | --- | --- | --- |
| **Well No.** |  | **µS/Cm** | **mg/L** | **mg/L** | **mgCaCO_3_/L** | **mg/L** | **mg/L** | **mg/L** | **mg/L** | **mg/L** | **mg/L** |
| 1 | 7.2 | 1368 | 848 | 390 | 468 | 213 | 88 | 0.4 | <0.05 | <0.05 | 21 |
| 2 | 7.36 | 1612 | 999 | 367 | 440 | 262 | 67 | 0.6 | <0.05 | <0.05 | 36 |
| 3 | 7.38 | 1136 | 704 | 197 | 238 | 168 | 41 | 0.4 | <0.05 | <0.05 | 9 |
| 4 | 7.36 | 1861 | 1154 | 205 | 246 | 315 | 161 | 1.5 | <0.05 | <0.05 | 9 |
| 5 | 6.94 | 10230 | 6343 | 281 | 302 | 3235 | 114 | 0.6 | <0.05 | <0.05 | 21 |
| 6 | 7.53 | 1430 | 887 | 225 | 282 | 241 | 72 | 0.4 | <0.05 | <0.05 | 19 |
| 7 | 7.46 | 1153 | 715 | 273 | 286 | 150 | 69 | 0.8 | <0.05 | <0.05 | 14 |
| 8 | 7.46 | 1463 | 907 | 309 | 354 | 269 | 53 | 0.8 | <0.05 | <0.05 | 44 |
| 9 | 7.34 | 2010 | 1246 | 336 | 650 | 395 | 60 | 0.9 | <0.05 | <0.05 | 53 |
| 10 | 7.29 | 2730 | 1693 | 433 | 578 | 532 | 159 | 0.7 | <0.05 | <0.05 | 80 |
| 11 | 7.27 | 1305 | 809 | 291 | 554 | 168 | 181 | 0.8 | <0.05 | <0.05 | 41 |
| 12 | 7.17 | 1399 | 867 | 309 | 506 | 189 | 164 | 0.8 | <0.05 | <0.05 | 62 |
| 13 | 6.94 | 10230 | 6343 | 261 | 400 | 3235 | 114 | 0.8 | <0.05 | <0.05 | 38 |
| 14 | 7.36 | 1227 | 761 | 276 | 282 | 182 | 16 | 1.1 | <0.05 | <0.05 | 16 |
| 15 | 7.12 | 1134 | 703 | 213 | 388 | 150 | 102 | 0.8 | <0.05 | <0.05 | 46 |
| 16 | 7.38 | 1310 | 812 | 339 | 200 | 161 | 63 | 1.1 | <0.05 | <0.05 | 25 |
| 17 | 7.62 | 1443 | 895 | 326 | 188 | 245 | 37 | 0.9 | <0.05 | <0.05 | 30 |
| 18 | 7.55 | 917 | 569 | 301 | 196 | 126 | 39 | 0.7 | <0.05 | <0.05 | 19 |
| 19 | 7.45 | 1380 | 856 | 268 | 282 | 234 | 76 | 0.8 | <0.05 | <0.05 | 19 |
| 20 | 7.12 | 1134 | 703 | 238 | 416 | 150 | 102 | 0.6 | <0.05 | <0.05 | 50 |
| 21 | 7.71 | 1303 | 808 | 273 | 390 | 196 | 115 | 0.7 | <0.05 | <0.05 | 32 |
| 22 | 7.74 | 1292 | 801 | 248 | 312 | 217 | 53 | 1.1 | <0.05 | <0.05 | 18 |
| 23 | 7 | 5910 | 3664 | 319 | 218 | 1654 | 138 | 1 | <0.05 | <0.05 | 41 |
| 24 | 7.39 | 1410 | 874 | 286 | 438 | 238 | 95 | 1.1 | <0.05 | <0.05 | 43 |
| 25 | 7 | 1816 | 1126 | 344 | 590 | 241 | 247 | 0.6 | <0.05 | <0.05 | 79 |
| 26 | 7.76 | 957 | 593 | 304 | 336 | 133 | 32 | 0.7 | <0.05 | <0.05 | 20 |
| 27 | 7.56 | 1522 | 944 | 473 | 300 | 273 | 42 | 0.4 | 76 | 73 | 1538 |
| 28 | 7.5 | 1490 | 924 | 250 | 298 | 238 | 128 | 0.8 | <0.05 | <0.05 | 28 |
| 29 | 7.58 | 803 | 498 | 190 | 200 | 91 | 34 | 0.7 | <0.05 | <0.05 | 8 |
| 30 | 7.28 | 1720 | 1066 | 299 | 420 | 308 | 60 | 0.6 | <0.05 | <0.05 | 58 |
| 31 | 6.99 | 11740 | 7279 | 336 | 504 | 3754 | 67 | 0.2 | <0.05 | <0.05 | 104 |
| 32 | 6.74 | 16350 | 10137 | 334 | 1256 | 6098 | 56 | 0.2 | 10 | <0.05 | 280 |
| 33 | 7.22 | 5460 | 3385 | 304 | 1258 | 1511 | 109 | 2.5 | 7 | <0.05 | 258 |
| 34 | 6.65 | 21000 | 13020 | 263 | 770 | 6784 | 139 | 0.5 | 2 | <0.05 | 80 |
| 35 | 7.02 | 9290 | 5760 | 243 | 552 | 2742 | 57 | 0.6 | <0.05 | <0.05 | 42 |
| 36 | 6.77 | 9370 | 5809 | 245 | 1720 | 2988 | 133 | 0.9 | 11 | <0.05 | 350 |
| 37 | 7.13 | 8270 | 5127 | 347 | 496 | 2320 | 103 | 0.7 | <0.05 | <0.05 | 187 |
| 38 | 7.13 | 8480 | 5258 | 463 | 508 | 2214 | 139 | 1.3 | 2 | <0.05 | 177 |
| 39 | 6.97 | 18810 | 11662 | 423 | 550 | 5800 | 82 | 1.3 | <0.05 | <0.05 | 167 |
| 40 | 7.16 | 15560 | 9647 | 225 | 2434 | 4921 | 60 | 0.9 | 19 | <0.05 | 196 |
| 41 | 7.3 | 29600 | 18352 | 314 | 450 | 9491 | 43 | 0.9 | <0.05 | <0.05 | 56 |
| 42 | 6.97 | 13350 | 8277 | 271 | 368 | 4237 | 65 |  |  |  |  |
| 43 | 6.95 | 9808 | 6081 | 288 | 410 | 2205 | 66 | 0.6 | <0.05 | <0.05 | 43 |
| 44 | 7.3 | 1290 | 800 | 304 | 426 | 158 | 158 | 0.6 | <0.05 | <0.05 | 38 |
| 45 | 7.27 | 1063 | 659 | 273 | 398 | 103 | 159 | 0.7 | 0.1 | <0.05 | 31 |
| 46 | 7.3 | 1111 | 682 | 271 | 356 | 110 | 156 | 1.1 | <0.05 | <0.05 | 35 |
| 47 | 6.74 | 16350 | 10137 | 362 | 1328 | 6098 | 56 | 0.5 | 0.7 | <0.05 | 396 |
| 48 | 7.51 | 7620 | 4724 | 390 | 316 | 2250 | 63 | 0.6 | <0.05 | <0.05 | 115 |
| 49 | 7.39 | 2320 | 1738 | 266 | 296 | 450 | 97 | 0.2 | 0.0 | <0.05 | 75 |
| 50 | 7.34 | 8730 | 5413 | 281 | 230 | 2584 | 56 | 1.3 | <0.05 | <0.05 | 80 |
| 51 | 7.28 | 8260 | 5121 | 253 | 242 | 2390 | 54 | 0.3 | <0.05 | <0.05 | 44 |
| 52 | 7.58 | 1900 | 1178 | 344 | 420 | 380 | 70 | 1.1 | 0.3 | <0.05 | 160 |
| 53 | 7.03 | 10680 | 6622 | 471 | 396 | 3164 | 150 | 0.8 | <0.05 | <0.05 | 222 |
| 54 | 7.38 | 1310 | 812 | 329 | 774 | 161 | 63 | 0.7 | <0.05 | <0.05 | 125 |
| 55 | 7.55 | 917 | 569 | 326 | 1160 | 126 | 39 | 0.8 | 0.4 | <0.05 | 215 |
| 56 | 7.62 | 1443 | 895 | 268 | 500 | 245 | 37 | 0.6 | <0.05 | <0.05 | 75 |
| 57 | 7.4 | 3360 | 2083 | 529 | 414 | 620 | 98 | 0.2 | <0.05 | <0.05 | 247 |
| 58 | 7.26 | 2940 | 1823 | 509 | 402 | 496 | 30 | 0.5 | <0.05 | <0.05 | 236 |
| 59 | 7.57 | 3410 | 2114 | 557 | 590 | 689 | 40 | 1.6 | 0.1 | <0.05 | 313 |
| 60 | 7.43 | 4434 | 2749 | 526 | 306 | 896 | 122 | 0.5 | 0.1 | <0.05 | 221 |
| 61 | 7.56 | 1522 | 944 | 362 | 278 | 273 | 42 | 1.1 | <0.05 | <0.05 | 46 |
| 62 | 7.3 | 5420 | 3360 | 261 | 290 | 1357 | 52 | 0.9 | 0.0 | <0.05 | 115 |
| 63 | 7.6 | 4730 | 4730 | 288 | 472 | 1111 | 51 | 1.1 | <0.05 | <0.05 | 326 |
| 64 | 7.7 | 4380 | 2803 | 253 | 458 | 1114 | 50 | 0.8 | <0.05 | <0.05 | 159 |
| 65 | 7.3 | 3330 | 2131.2 | 311 | 308 | 864 | 68 | 1.2 | <0.05 | <0.05 | 240 |
| 66 | 7.67 | 2800 | 1792 | 263 | 320 | 689 | 41 | 1.3 | <0.05 | <0.05 | 179 |
| 67 | 7.86 | 4790 | 3065.6 | 293 | 706 | 1088 | 196 | 1.4 | <0.05 | <0.05 | 421 |
| 68 | 8.08 | 3220 | 2060.8 | 276 | 336 | 788 | 73 | 1.1 | 0.1 | <0.05 | 244 |
| 69 | 7.83 | 5050 | 3232 | 258 | 530 | 1251 | 114 | 1.1 | <0.05 | <0.05 | 299 |
| 70 | 7.5 | 15540 | 9635 | 478 | 852 | 6610 | 93 | 1.4 | 0.3 | <0.05 | 462 |
| 71 | 7.86 | 5130 | 3283.2 | 218 | 368 | 1212 | 111 | 1.5 | 0.1 | <0.05 | 172 |
| 72 | 7.85 | 23500 | 15040 | 240 | 582 | 8819 | 34 | 0.9 | <0.05 | <0.05 | 330 |
| 73 | 7.86 | 1818 | 1127 | 239 | 400 | 281 | 107 | 0.8 | <0.05 | <0.05 | 237 |
| 74 | 7.98 | 4380 | 2416 | 266 | 430 | 810 | 75 | 1.1 | <0.05 | <0.05 | 197 |
| 75 | 7.01 | 4550 | 2821 | 261 | 1066 | 978 | 107 | 1.5 | 0.4 | <0.05 | 421 |
| 76 | 7.43 | 3870 | 2399 | 266 | 804 | 922 | 62 | 1.3 | 0.3 | <0.05 | 467 |
| 77 | 8.07 | 2660 | 1702.4 | 202 | 390 | 699 | 56 | 1.4 | 0.2 | <0.05 | 218 |
| 78 | 7.84 | 3800 | 2432 | 253 | 488 | 849 | 61 | 1.5 | 0.2 | <0.05 | 259 |
| 79 | 7.87 | 3910 | 2502.4 | 400 | 290 | 928 | 35 |  |  |  |  |
| 80 | 7.75 | 14990 | 9593.6 | 349 | 610 | 4602 | 123 | 1.4 | 0.3 | <0.05 | 416 |
| 81 | 7.65 | 4440 | 2841.6 | 273 | 308 | 1035 | 63 | 1.1 | <0.05 | <0.05 | 64 |
| 82 | 7.3 | 5390 | 3449.6 | 218 | 208 | 1252 | 184 | 1.3 | <0.05 | <0.05 | 87 |
| 83 | 7.6 | 4090 | 2618 | 273 | 668 | 877 | 297 | 2.1 | <0.05 | <0.05 | 264 |
| 84 | 7.9 | 709 | 355 | 218 | 500 | 123 | 76 | 2.4 | 0.4 | <0.05 | 250 |
| 85 | 7.6 | 1109 | 688 | 364 | 254 | 199 | 63 | 1.6 | 0.2 | <0.05 | 396 |
| 86 | 7.2 | 5440 | 3482 | 468 | 828 | 1160 | 364 | 1.2 | <0.05 | <0.05 | 226 |
| 87 | 7.9 | 4580 | 2840 | 359 | 358 | 927 | 264 | 1.7 | 0.2 | <0.05 | 494 |
| 88 | 7.8 | 5630 | 3603 | 329 | 210 | 1431 | 246 | 1.2 | <0.05 | <0.05 | 163 |
| 89 | 7.8 | 8340 | 5338 | 407 | 480 | 2237 | 215 | 1.3 | 0.5 | <0.05 | 621 |
| 90 | 7.8 | 3110 | 1990 | 164 | 398 | 770 | 281 | 1.1 | <0.05 | <0.05 | 159 |
| 91 | 7.1 | 3750 | 2325 | 301 | 588 | 867 | 365 |  |  |  |  |
| 92 | 7.6 | 8070 | 5003 | 331 | 710 | 2074 | 108 | 1.2 | <0.05 | <0.05 | 121 |
| 93 | 7.6 | 3540 | 2266 | 230 | 744 | 759 | 338 | 1.2 | <0.05 | <0.05 | 161 |
| 94 | 7.7 | 1838 | 1140 | 235 | 280 | 278 | 165 | 1.3 | 0.1 | <0.05 | 46 |
| 95 | 8.0 | 4380 | 2416 | 301 | 600 | 810 | 75 | 1.4 | 0.4 | <0.05 | 483 |
| 96 | 7.8 | 5630 | 3603 | 175 | 310 | 1431 | 246 | 2 | 0.1 | <0.05 | 173 |
| 97 | 7.8 | 7870 | 5037 | 182 | 346 | 2199 | 148 | 1.5 | <0.05 | <0.05 | 203 |
| 98 | 7.8 | 8340 | 5338 | 164 | 506 | 2237 | 215 | 1.2 | <0.05 | <0.05 | 176 |
| 99 | 7.8 | 4860 | 3013 | 342 | 386 | 1026 | 74 | 1.7 | 0.4 | <0.05 | 659 |
| 100 | 7.8 | 1009 | 505 | 382 | 224 | 178 | 127 | 2.1 | 0.2 | <0.05 | 416 |
| 101 | 7.5 | 3280 | 2034 | 425 | 104 | 631 | 216 | 2.2 | <0.05 | <0.05 | 79 |
| 102 | 7.7 | 5630 | 3491 | 185 | 82 | 1497 | 108 | 2.3 | <0.05 | <0.05 | 46 |
| 103 | 7.7 | 4460 | 2765 | 149 | 160 | 1160 | 72 | 2.5 | <0.05 | <0.05 | 46 |
| 104 | 7.5 | 5920 | 3670 | 197 | 138 | 1554 | 143 | 2.6 | <0.05 | <0.05 | 96 |
| 105 | 7.6 | 2540 | 1626 | 276 | 464 | 700 | 89 | 1.2 | 0.4 | <0.05 | 143 |
| 106 | 7.7 | 5110 | 3168 | 319 | 326 | 999 | 235 | 1.3 | <0.05 | <0.05 | 142 |
| 107 | 7.6 | 1586 | 983 | 132 | 102 | 345 | 120 | 2 | <0.05 | <0.05 | 75 |
| 108 | 7.5 | 2708 | 1679 | 142 | 210 | 723 | 118 | 1.4 | <0.05 | <0.05 | 15 |
| 109 | 7.4 | 5710 | 3540 | 185 | 366 | 1302 | 229 | 1.2 | 0.2 | <0.05 | 528 |
| 110 | 7.4 | 6690 | 4148 | 182 | 550 | 1633 | 71 | 2.2 | 0.2 | <0.05 | 117 |
| 111 | 7.5 | 2870 | 1779 | 205 | 698 | 630 | 146 | 1.7 | 0.3 | <0.05 | 515 |
| 112 | 7.6 | 5011 | 3107 | 311 | 570 | 1089 | 192 | 1.6 | 0.2 | <0.05 | 293 |
| 113 | 7.5 | 3360 | 2083 | 142 | 164 | 737 | 227 | 1.4 | <0.05 | <0.05 | 49 |
| 114 | 7.5 | 3340 | 2071 | 182 | 120 | 772 | 178 | 1.3 | <0.05 | <0.05 | 41 |
| 115 | 7.7 | 9600 | 5952 | 202 | 184 | 2136 | 83 | 1.2 | 0.0 | <0.05 | 169 |

**S2:** Results of Major Cations

|  | **Ca** | **Mg** | **Na** | **K** |
| --- | --- | --- | --- | --- |
| **Well No.** | **mg/L** | **mg/L** | **mg/L** | **mg/L** |
| 1 | 106 | 49 | 66.2 | 2.15 |
| 2 | 106 | 47 | 57.8 | 5.64 |
| 3 | 45 | 29 | 27.4 | 2.87 |
| 4 | 56 | 26 | 28.7 | 1.62 |
| 5 | 74 | 28 | 40.4 | 4.85 |
| 6 | 52 | 34 | 46.1 | 2.78 |
| 7 | 48 | 34 | 58 | 2.51 |
| 8 | 53 | 49 | 135 | 2.86 |
| 9 | 102 | 86 | 215 | 4.08 |
| 10 | 97 | 79 | 210 | 4.03 |
| 11 | 124 | 56 | 66.8 | 2.81 |
| 12 | 123 | 50 | 94.1 | 5.89 |
| 13 | 89 | 40 | 58.4 | 5.48 |
| 14 | 49 | 39 | 60.8 | 3.69 |
| 15 | 78 | 53 | 37.7 | 1.85 |
| 16 | 31 | 31 | 125 | 2.49 |
| 17 | 26 | 27 | 130 | 2.3 |
| 18 | 29 | 29 | 87.7 | 2.19 |
| 19 | 53 | 35 | 68.8 | 3.47 |
| 20 | 89 | 43 | 77.2 | 2.48 |
| 21 | 66 | 59 | 89.6 | 3.42 |
| 22 | 58 | 37 | 39.4 | 2.77 |
| 23 | 89 | 38 | 58 | 6.13 |
| 24 | 77 | 65 | 42.8 | 2.08 |
| 25 | 117 | 71 | 101 | 9.07 |
| 26 | 26 | 28 | 90.5 | 1.98 |
| 27 | 57 | 41 | 119 | 10.7 |
| 28 | 67 | 31 | 60.5 | 3.52 |
| 29 | 38 | 17 | 22.3 | 1.17 |
| 30 | 76 | 55 | 94.3 | 2.32 |
| 31 | 105 | 54 | 210 | 4.6 |
| 32 | 170 | 183 | 380 | 18 |
| 33 | 260 | 136 | 330 | 8.79 |
| 34 | 175 | 85 | 230 | 7.07 |
| 35 | 152 | 43 | 125 | 4.08 |
| 36 | 340 | 190 | 420 | 19 |
| 37 | 107 | 79 | 270 | 20 |
| 38 | 80 | 65 | 260 | 29 |
| 39 | 82 | 71 | 260 | 9.57 |
| 40 | 375 | 400 | 365 | 14 |
| 41 | 83 | 49 | 101 | 6.16 |
| 42 | 75 | 39 | 83.7 | 2.76 |
| 43 | 98 | 37 | 59.5 | 3.73 |
| 44 | 107 | 42 | 49.6 | 6.45 |
| 45 | 100 | 38 | 36.1 | 5.79 |
| 46 | 92 | 31 | 51.9 | 7.15 |
| 47 | 192 | 198 | 430 | 48 |
| 48 | 52 | 34 | 221 | 4.42 |
| 49 | 58 | 30 | 150 | 2.47 |
| 50 | 28 | 34 | 160 | 3.09 |
| 51 | 51 | 24 | 92.2 | 1.69 |
| 52 | 67 | 59 | 240 | 4.88 |
| 53 | 45 | 55 | 280 | 5.68 |
| 54 | 198 | 66 | 240 | 15 |
| 55 | 280 | 110 | 315 | 14 |
| 56 | 125 | 42 | 160 | 2.59 |
| 57 | 46 | 57 | 290 | 10.2 |
| 58 | 53 | 61 | 285 | 26 |
| 59 | 73 | 84 | 303 | 24 |
| 60 | 33 | 44 | 280 | 5.75 |
| 61 | 46 | 39 | 138 | 3.84 |
| 62 | 37 | 39 | 191 | 3.06 |
| 63 | 66 | 66 | 268 | 4.82 |
| 64 | 102 | 43 | 180 | 5.81 |
| 65 | 44 | 43 | 244 | 4.65 |
| 66 | 50 | 40 | 231 | 4.53 |
| 67 | 113 | 99 | 251 | 13 |
| 68 | 56 | 47 | 240 | 5.75 |
| 69 | 102 | 69 | 200 | 5.23 |
| 70 | 117 | 111 | 320 | 32 |
| 71 | 68 | 48 | 126 | 3.02 |
| 72 | 112 | 68 | 130 | 4.29 |
| 73 | 50 | 57 | 221 | 6.41 |
| 74 | 90 | 45 | 161 | 7.33 |
| 75 | 170 | 133 | 290 | 35 |
| 76 | 135 | 108 | 299 | 9.61 |
| 77 | 57 | 43 | 267 | 4.82 |
| 78 | 75 | 59 | 287 | 5.54 |
| 79 | 47 | 32 | 91 | 1.65 |
| 80 | 96 | 75 | 295 | 8.72 |
| 81 | 41 | 44 | 164 | 2.6 |
| 82 | 22 | 29 | 187 | 2.48 |
| 83 | 107 | 88 | 270 | 9.3 |
| 84 | 67 | 69 | 320 | 19 |
| 85 | 34 | 30 | 284 | 5.31 |
| 86 | 128 | 117 | 253 | 9.37 |
| 87 | 50 | 47 | 246 | 8.58 |
| 88 | 27 | 27 | 241 | 4.07 |
| 89 | 71 | 68 | 387 | 23 |
| 90 | 68 | 55 | 230 | 5.98 |
| 91 | 127 | 63 | 164 | 5.73 |
| 92 | 177 | 78 | 130 | 15 |
| 93 | 131 | 95 | 241 | 5.74 |
| 94 | 39 | 44 | 98.8 | 2.15 |
| 95 | 90 | 78 | 321 | 9.91 |
| 96 | 54 | 42 | 200 | 3.74 |
| 97 | 60 | 46 | 205 | 4.5 |
| 98 | 93 | 66 | 206 | 4.69 |
| 99 | 48 | 50 | 352 | 8.62 |
| 100 | 28 | 28 | 321 | 6.06 |
| 101 | 13 | 11 | 271 | 2.36 |
| 102 | 15 | 11 | 108 | 1.93 |
| 103 | 29 | 20 | 122 | 2.85 |
| 104 | 22 | 17 | 130 | 2.77 |
| 105 | 83 | 67 | 200 | 27 |
| 106 | 49 | 42 | 330 | 7.58 |
| 107 | 18 | 11 | 80.3 | 2.21 |
| 108 | 36 | 28 | 175 | 4.78 |
| 109 | 64 | 45 | 189 | 4.29 |
| 110 | 99 | 67 | 176 | 5.07 |
| 111 | 139 | 84 | 103 | 7.15 |
| 112 | 95 | 73 | 160 | 14 |
| 113 | 33 | 22 | 91.2 | 2.73 |
| 114 | 20 | 12 | 80.7 | 1.93 |
| 115 | 31 | 23 | 200 | 3.48 |

**S3:** Results of Metals and Metalloids

|  | Ag | Al | As | Ba | Cd | Co | Cr | Cu | Fe | Mn | Ni | Pb | Sr | Sr | Zn |
| --- | --- | --- | --- | --- | --- | --- | --- | --- | --- | --- | --- | --- | --- | --- | --- |
| Well No. | µg/L | µg/L | µg/L | µg/L | µg/L | µg/L | µg/L | µg/L | µg/L | µg/L | µg/L | µg/L | µg/L | mg/L | µg/L |
| 1 | <1.7 | <0.02 | <16.7 | 417 | <0.3 | <1.7 | 8 | <3.3 | 91 | 1.28 | <16.7 | <3.3 | 1760 | 1.8 | 14.7 |
| 2 | <1.7 | <0.02 | <16.7 | 197 | <0.3 | <1.7 | 10.1 | <3.3 | 94 | 3.75 | <16.7 | <3.3 | 1500 | 1.5 | 8.75 |
| 3 | <1.7 | <0.02 | <16.7 | 343 | <0.3 | <1.7 | 10.3 | <3.3 | 89 | 2.8 | <16.7 | <3.3 | 1110 | 1.1 | 10.9 |
| 4 | <1.7 | <0.02 | <16.7 | 306 | <0.3 | <1.7 | 15.3 | <3.3 | 106 | 2.17 | <16.7 | <3.3 | 1020 | 1.0 | 8.68 |
| 5 | <1.7 | <0.02 | <16.7 | 260 | <0.3 | <1.7 | 17.9 | <3.3 | 78 | 1.06 | <16.7 | <3.3 | 1200 | 1.2 | 8.55 |
| 6 | <1.7 | <0.02 | <16.7 | 198 | <0.3 | <1.7 | 11.4 | <3.3 | 102 | 1.39 | <16.7 | <3.3 | 1220 | 1.2 | 16.2 |
| 7 | <1.7 | <0.02 | <16.7 | 179 | <0.3 | <1.7 | 17.5 | <3.3 | 98 | 1.57 | <16.7 | <3.3 | 1060 | 1.1 | 6.51 |
| 8 | <1.7 | <0.02 | <16.7 | 119 | <0.3 | <1.7 | BDL | <3.3 | 1 | 0.07 | <16.7 | <3.3 | 1160 | 1.2 | 1.42 |
| 9 | <1.7 | <0.02 | <16.7 | 203 | <0.3 | <1.7 | 13.5 | <3.3 | 127 | 1.91 | <16.7 | <3.3 | 2120 | 2.1 | 9.5 |
| 10 | <1.7 | <0.02 | <16.7 | 228 | <0.3 | <1.7 | 13.6 | <3.3 | 95 | 1.91 | <16.7 | <3.3 | 2090 | 2.1 | 6.92 |
| 11 | <1.7 | <0.02 | <16.7 | 233 | <0.3 | <1.7 | 12.9 | <3.3 | 119 | 2.43 | <16.7 | <3.3 | 2180 | 2.2 | 61.2 |
| 12 | <1.7 | <0.02 | <16.7 | 173 | <0.3 | <1.7 | 7.98 | <3.3 | 1 | 0.07 | <16.7 | <3.3 | 1850 | 1.9 | 25.4 |
| 13 | <1.7 | <0.02 | <16.7 | 254 | <0.3 | <1.7 | 17.7 | <3.3 | 104 | 2.6 | <16.7 | <3.3 | 1670 | 1.7 | 16.6 |
| 14 | <1.7 | <0.02 | <16.7 | 214 | <0.3 | <1.7 | 11.3 | <3.3 | 104 | 2.31 | <16.7 | <3.3 | 1200 | 1.2 | 11.4 |
| 15 | <1.7 | <0.02 | <16.7 | 205 | <0.3 | <1.7 | 14.3 | <3.3 | 88 | 1.22 | <16.7 | <3.3 | 1780 | 1.8 | 5.58 |
| 16 | <1.7 | <0.02 | <16.7 | 17.9 | <0.3 | <1.7 | 10.7 | <3.3 | 0 | 0.10 | <16.7 | <3.3 | 818 | 0.8 | 5.11 |
| 17 | <1.7 | <0.02 | <16.7 | 43.6 | <0.3 | <1.7 | 17.3 | <3.3 | 121 | 1.88 | <16.7 | <3.3 | 645 | 0.6 | 8.05 |
| 18 | <1.7 | <0.02 | <16.7 | 55.3 | <0.3 | <1.7 | 15.8 | <3.3 | 99 | 1.31 | <16.7 | <3.3 | 742 | 0.7 | 7.33 |
| 19 | <1.7 | <0.02 | <16.7 | 174 | <0.3 | <1.7 | 12.3 | <3.3 | 94 | 1.56 | <16.7 | <3.3 | 1220 | 1.2 | 23.2 |
| 20 | <1.7 | <0.02 | <16.7 | 346 | <0.3 | <1.7 | 17.6 | <3.3 | 128 | 1.64 | <16.7 | <3.3 | 1850 | 1.9 | 6.77 |
| 21 | <1.7 | <0.02 | <16.7 | 147 | <0.3 | <1.7 | 8.47 | <3.3 | 0 | BDL | <16.7 | <3.3 | 1670 | 1.7 | 6.8 |
| 22 | <1.7 | <0.02 | <16.7 | 208 | <0.3 | <1.7 | 12.8 | <3.3 | 98 | 1.34 | <16.7 | <3.3 | 1320 | 1.3 | 7.22 |
| 23 | <1.7 | <0.02 | <16.7 | 186 | <0.3 | <1.7 | 12.8 | <3.3 | 1 | 0.449 | <16.7 | <3.3 | 1340 | 1.3 | 10.7 |
| 24 | <1.7 | <0.02 | <16.7 | 158 | <0.3 | <1.7 | 7.85 | <3.3 | 0 | 0.0614 | <16.7 | <3.3 | 2140 | 2.1 | 8.71 |
| 25 | <1.7 | <0.02 | <16.7 | 173 | <0.3 | <1.7 | 12.7 | <3.3 | 92 | 1.65 | <16.7 | <3.3 | 2360 | 2.4 | 15.9 |
| 26 | <1.7 | <0.02 | <16.7 | 50.3 | <0.3 | <1.7 | 16.7 | <3.3 | 106 | 1.9 | <16.7 | <3.3 | 556 | 0.6 | 11.6 |
| 27 | <1.7 | <0.02 | <16.7 | 159 | <0.3 | <1.7 | 14.5 | <3.3 | 96 | 1.24 | <16.7 | <3.3 | 1300 | 1.3 | 7.59 |
| 28 | <1.7 | <0.02 | <16.7 | 219 | <0.3 | <1.7 | 12.7 | <3.3 | 117 | 1.52 | <16.7 | <3.3 | 1230 | 1.2 | 7.1 |
| 29 | <1.7 | <0.02 | <16.7 | 53.9 | <0.3 | <1.7 | 7.48 | <3.3 | 0 | 0.208 | <16.7 | <3.3 | 721 | 0.7 | 3 |
| 30 | <1.7 | <0.02 | <16.7 | 179 | <0.3 | <1.7 | 8.5 | <3.3 | 99 | 1.77 | <16.7 | <3.3 | 1480 | 1.5 | 8.78 |
| 31 | <1.7 | <0.02 | <16.7 | 172 | <0.3 | <1.7 | 22.9 | <3.3 | 89 | 1.21 | <16.7 | <3.3 | 3280 | 3.3 | 9.02 |
| 32 | <1.7 | <0.02 | <16.7 | 131 | <0.3 | <1.7 | 16.1 | <3.3 | 11 | 0.359 | <16.7 | <3.3 | 5180 | 5.2 | 2.25 |
| 33 | <1.7 | <0.02 | <16.7 | 194 | <0.3 | <1.7 | 22.3 | <3.3 | 76 | 1.27 | <16.7 | <3.3 | 7790 | 7.8 | 9.33 |
| 34 | <1.7 | <0.02 | <16.7 | 330 | <0.3 | <1.7 | 18.9 | <3.3 | 79 | 1.51 | <16.7 | <3.3 | 5400 | 5.4 | 5.39 |
| 35 | <1.7 | <0.02 | <16.7 | 416 | <0.3 | <1.7 | 18.3 | <3.3 | 90 | 1.39 | <16.7 | <3.3 | 4360 | 4.4 | 7.73 |
| 36 | <1.7 | <0.02 | <16.7 | 352 | <0.3 | <1.7 | 9.54 | <3.3 | 6 | 0.431 | <16.7 | <3.3 | 9640 | 9.6 | 2.65 |
| 37 | <1.7 | <0.02 | <16.7 | 96.6 | <0.3 | <1.7 | 15.1 | <3.3 | 0 | 0.138 | <16.7 | <3.3 | 3010 | 3.0 | 4.6 |
| 38 | <1.7 | <0.02 | <16.7 | 65.9 | <0.3 | <1.7 | 25.4 | <3.3 | 72 | 0.967 | <16.7 | <3.3 | 2070 | 2.1 | 8.39 |
| 39 | <1.7 | <0.02 | <16.7 | 76.2 | <0.3 | <1.7 | 23.6 | <3.3 | 80 | 1.1 | <16.7 | <3.3 | 2380 | 2.4 | 15.6 |
| 40 | <1.7 | <0.02 | <16.7 | 344 | <0.3 | <1.7 | 13.7 | <3.3 | 102 | 1.69 | <16.7 | <3.3 | 9390 | 9.4 | 6.79 |
| 41 | <1.7 | <0.02 | <16.7 | 216 | <0.3 | <1.7 | 17.9 | <3.3 | 89 | 1.37 | <16.7 | <3.3 | 1700 | 1.7 | 8.83 |
| 42 | <1.7 | <0.02 | <16.7 | 239 | <0.3 | <1.7 | 16.1 | <3.3 | 86 | 1.28 | <16.7 | <3.3 | 1480 | 1.5 | 7.08 |
| 43 | <1.7 | <0.02 | <16.7 | 220 | <0.3 | <1.7 | 16.2 | <3.3 | 97 | 1.21 | <16.7 | <3.3 | 1470 | 1.5 | 7.8 |
| 44 | <1.7 | <0.02 | <16.7 | 209 | <0.3 | <1.7 | 10.8 | <3.3 | 78 | 1.07 | <16.7 | <3.3 | 1480 | 1.5 | 9.99 |
| 45 | <1.7 | <0.02 | <16.7 | 210 | <0.3 | <1.7 | 6.9 | <3.3 | 0 | 0.193 | <16.7 | <3.3 | 1340 | 1.3 | 3.01 |
| 46 | <1.7 | <0.02 | <16.7 | 204 | <0.3 | <1.7 | 9.38 | <3.3 | 0 | 0.071 | <16.7 | <3.3 | 1100 | 1.1 | 9.03 |
| 47 | <1.7 | <0.02 | <16.7 | 117 | <0.3 | <1.7 | 25.5 | <3.3 | 1 | 0.196 | <16.7 | <3.3 | 6960 | 7.0 | 4.6 |
| 48 | <1.7 | <0.02 | <16.7 | 118 | <0.3 | <1.7 | 37.6 | <3.3 | 91 | 1.17 | <16.7 | <3.3 | 1640 | 1.6 | 7.27 |
| 49 | <1.7 | <0.02 | <16.7 | 244 | <0.3 | <1.7 | 25.3 | <3.3 | 81 | 1.14 | <16.7 | <3.3 | 2590 | 2.6 | 6.18 |
| 50 | <1.7 | <0.02 | <16.7 | 134 | <0.3 | <1.7 | 31.5 | <3.3 | 95 | 1.77 | <16.7 | <3.3 | 1060 | 1.1 | 8.38 |
| 51 | <1.7 | <0.02 | <16.7 | 306 | <0.3 | <1.7 | 24.1 | <3.3 | 76 | 1.07 | <16.7 | <3.3 | 1840 | 1.8 | 6.27 |
| 52 | <1.7 | <0.02 | <16.7 | 199 | <0.3 | <1.7 | 27.7 | <3.3 | 81 | 1.3 | <16.7 | <3.3 | 2280 | 2.3 | 6.99 |
| 53 | <1.7 | <0.02 | <16.7 | 50.2 | <0.3 | <1.7 | 27.6 | <3.3 | 108 | 2.32 | <16.7 | <3.3 | 1520 | 1.5 | 6.14 |
| 54 | <1.7 | <0.02 | <16.7 | 156 | <0.3 | <1.7 | 19.1 | <3.3 | 102 | 1.95 | <16.7 | <3.3 | 5450 | 5.5 | 17.5 |
| 55 | <1.7 | <0.02 | <16.7 | 124 | <0.3 | <1.7 | 17.7 | <3.3 | 98 | 1.84 | <16.7 | <3.3 | 6680 | 6.7 | 57.3 |
| 56 | <1.7 | <0.02 | <16.7 | 180 | <0.3 | <1.7 | 21.3 | <3.3 | 102 | 1.87 | <16.7 | <3.3 | 4820 | 4.8 | 35.6 |
| 57 | <1.7 | <0.02 | <16.7 | 64.1 | <0.3 | <1.7 | 24.5 | <3.3 | 87 | 2.1 | <16.7 | <3.3 | 1430 | 1.4 | 8.03 |
| 58 | <1.7 | <0.02 | <16.7 | 49.4 | <0.3 | <1.7 | 18.9 | <3.3 | 86 | 1.1 | <16.7 | <3.3 | 1590 | 1.6 | 7.78 |
| 59 | <1.7 | <0.02 | <16.7 | 64.8 | <0.3 | <1.7 | 31.8 | <3.3 | 85 | 1.05 | <16.7 | <3.3 | 2160 | 2.2 | 7.68 |
| 60 | <1.7 | <0.02 | <16.7 | 66.1 | <0.3 | <1.7 | 22.3 | <3.3 | 85 | 1.22 | <16.7 | <3.3 | 1080 | 1.1 | 16.3 |
| 61 | <1.7 | <0.02 | <16.7 | 118 | <0.3 | <1.7 | 11.4 | <3.3 | 87 | 1.37 | <16.7 | <3.3 | 1150 | 1.2 | 8.95 |
| 62 | <1.7 | <0.02 | <16.7 | 138 | <0.3 | <1.7 | 30.2 | <3.3 | 122 | 1.28 | <16.7 | <3.3 | 1770 | 1.8 | 9.24 |
| 63 | <1.7 | <0.02 | <16.7 | 149 | <0.3 | <1.7 | 21.3 | <3.3 | 118 | 1.97 | <16.7 | <3.3 | 1940 | 1.9 | 8.7 |
| 64 | <1.7 | <0.02 | <16.7 | 219 | <0.3 | <1.7 | 21.5 | <3.3 | 38 | 1.22 | <16.7 | <3.3 | 4160 | 4.2 | 19.6 |
| 65 | 1.9 | <0.02 | <16.7 | 59.8 | <0.3 | <1.7 | 22.4 | <3.3 | 7 | 0.553 | <16.7 | <3.3 | 1350 | 1.4 | 5 |
| 66 | 1.8 | <0.02 | <16.7 | 51.7 | <0.3 | <1.7 | 14.6 | <3.3 | 11 | 0.411 | <16.7 | <3.3 | 1390 | 1.4 | 5.5 |
| 67 | <1.7 | <0.02 | <16.7 | 64.5 | <0.3 | <1.7 | 45.9 | <3.3 | 1 | 0.146 | <16.7 | <3.3 | 4370 | 4.4 | 3.93 |
| 68 | <1.7 | <0.02 | <16.7 | 53.9 | <0.3 | <1.7 | 17.2 | <3.3 | 5 | 0.111 | <16.7 | <3.3 | 1570 | 1.6 | 1.74 |
| 69 | <1.7 | <0.02 | <16.7 | 56.3 | <0.3 | <1.7 | 29.3 | <3.3 | 1 | 2.16 | <16.7 | <3.3 | 4030 | 4.0 | 1.51 |
| 70 | 2.1 | <0.02 | <16.7 | 28.2 | <0.3 | <1.7 | 29.6 | <3.3 | 6 | 0.454 | <16.7 | <3.3 | 6260 | 6.3 | 14.1 |
| 71 | 1.7 | <0.02 | <16.7 | 100 | <0.3 | <1.7 | 28.3 | <3.3 | 8 | 0.467 | <16.7 | <3.3 | 3150 | 3.2 | 6.5 |
| 72 | 1.9 | <0.02 | <16.7 | 52 | <0.3 | <1.7 | 11 | <3.3 | 29 | 0.899 | <16.7 | <3.3 | 4560 | 4.6 | 3.41 |
| 73 | <1.7 | <0.02 | <16.7 | 97.6 | <0.3 | <1.7 | 33.1 | <3.3 | 8 | 0.834 | <16.7 | <3.3 | 2240 | 2.2 | 4.68 |
| 74 | 1.9 | <0.02 | <16.7 | 135 | <0.3 | <1.7 | 23.2 | <3.3 | 8 | 0.687 | <16.7 | <3.3 | 1950 | 2.0 | 5.88 |
| 75 | <1.7 | <0.02 | <16.7 | 29.5 | <0.3 | <1.7 | 26.3 | <3.3 | 9 | 0.505 | <16.7 | <3.3 | 12350 | 12.4 | 3.73 |
| 76 | <1.7 | <0.02 | <16.7 | 38.4 | <0.3 | <1.7 | 19.8 | <3.3 | 2 | 0.563 | <16.7 | <3.3 | 5250 | 5.3 | 11.7 |
| 77 | <1.7 | <0.02 | <16.7 | 57.2 | <0.3 | <1.7 | 13.8 | <3.3 | 10 | 0.507 | <16.7 | <3.3 | 1600 | 1.6 | 5.31 |
| 78 | <1.7 | <0.02 | <16.7 | 44.7 | <0.3 | <1.7 | 19 | <3.3 | 10 | 0.367 | <16.7 | <3.3 | 2180 | 2.2 | 17.7 |
| 79 | <1.7 | <0.02 | <16.7 | 237 | <0.3 | <1.7 | 8.81 | <3.3 | 22 | 1.79 | <16.7 | <3.3 | 1310 | 1.3 | 5.71 |
| 80 | <1.7 | <0.02 | <16.7 | 22.2 | <0.3 | <1.7 | 17.8 | <3.3 | 43 | 0.72 | <16.7 | <3.3 | 2700 | 2.7 | 13.6 |
| 81 | <1.7 | <0.02 | <16.7 | 219 | <0.3 | <1.7 | 15.8 | <3.3 | 0 | 0.107 | <16.7 | <3.3 | 1400 | 1.4 | 7.47 |
| 82 | <1.7 | <0.02 | <16.7 | 144 | <0.3 | <1.7 | 23.4 | <3.3 | 3 | 0.237 | <16.7 | <3.3 | 814 | 0.8 | 7.65 |
| 83 | <1.7 | <0.02 | <16.7 | 87.4 | <0.3 | <1.7 | 41.3 | <3.3 | 79 | 2 | <16.7 | <3.3 | 3970 | 4.0 | 8.62 |
| 84 | <1.7 | <0.02 | <16.7 | 50.6 | <0.3 | <1.7 | 58.3 | <3.3 | 13 | 0.828 | <16.7 | <3.3 | 1830 | 1.8 | 5.22 |
| 85 | <1.7 | <0.02 | <16.7 | 77.6 | <0.3 | <1.7 | 52.6 | <3.3 | 97 | 1.68 | <16.7 | <3.3 | 1150 | 1.2 | 21.7 |
| 86 | <1.7 | <0.02 | <16.7 | 60.9 | <0.3 | <1.7 | 36.5 | <3.3 | 93 | 1.12 | <16.7 | <3.3 | 5620 | 5.6 | 7.57 |
| 87 | <1.7 | <0.02 | <16.7 | 39.9 | <0.3 | <1.7 | 43.7 | <3.3 | 91 | 1.21 | <16.7 | <3.3 | 1730 | 1.7 | 7.52 |
| 88 | <1.7 | <0.02 | <16.7 | 244 | <0.3 | <1.7 | 50.2 | <3.3 | 94 | 1.29 | <16.7 | <3.3 | 1370 | 1.4 | 6.63 |
| 89 | <1.7 | <0.02 | <16.7 | 19.8 | <0.3 | <1.7 | 102 | <3.3 | 3 | 0.229 | <16.7 | <3.3 | 1800 | 1.8 | 6.19 |
| 90 | <1.7 | <0.02 | <16.7 | 140 | <0.3 | <1.7 | 29.7 | <3.3 | 1 | 0.127 | <16.7 | <3.3 | 2650 | 2.7 | 1.44 |
| 91 | <1.7 | <0.02 | <16.7 | 131 | <0.3 | <1.7 | 25.2 | <3.3 | 69 | 0.92 | <16.7 | <3.3 | 4240 | 4.2 | 7.04 |
| 92 | <1.7 | <0.02 | <16.7 | 109 | <0.3 | <1.7 | 18.6 | <3.3 | 1 | 0.137 | <16.7 | <3.3 | 6420 | 6.4 | 3.95 |
| 93 | <1.7 | <0.02 | <16.7 | 143 | <0.3 | <1.7 | 31.2 | <3.3 | 99 | 1.47 | <16.7 | <3.3 | 4880 | 4.9 | 18.8 |
| 94 | <1.7 | <0.02 | <16.7 | 188 | <0.3 | <1.7 | 24.7 | <3.3 | 5 | 0.659 | <16.7 | <3.3 | 1580 | 1.6 | 3.46 |
| 95 | <1.7 | <0.02 | <16.7 | 59 | <0.3 | <1.7 | 56.3 | <3.3 | 96 | 1.64 | <16.7 | <3.3 | 3130 | 3.1 | 19.6 |
| 96 | <1.7 | <0.02 | <16.7 | 112 | <0.3 | <1.7 | 42.3 | <3.3 | 3 | 0.125 | <16.7 | <3.3 | 2130 | 2.1 | 2.31 |
| 97 | <1.7 | <0.02 | <16.7 | 156 | <0.3 | <1.7 | 48 | <3.3 | 92 | 1.35 | <16.7 | <3.3 | 2220 | 2.2 | 7.86 |
| 98 | <1.7 | <0.02 | <16.7 | 73.9 | <0.3 | <1.7 | 37.7 | <3.3 | 86 | 1.2 | <16.7 | <3.3 | 3790 | 3.8 | 6.81 |
| 99 | <1.7 | <0.02 | <16.7 | 28.4 | <0.3 | <1.7 | 84.8 | <3.3 | 430 | 4.07 | <16.7 | <3.3 | 1870 | 1.9 | 5.81 |
| 100 | <1.7 | <0.02 | <16.7 | 34.3 | <0.3 | <1.7 | 52.8 | <3.3 | 105 | 1.83 | <16.7 | <3.3 | 1080 | 1.1 | 12.4 |
| 101 | <1.7 | <0.02 | <16.7 | 184 | <0.3 | <1.7 | 39.4 | <3.3 | 124 | 3.23 | <16.7 | <3.3 | 539 | 0.5 | 13.3 |
| 102 | <1.7 | <0.02 | <16.7 | 149 | <0.3 | <1.7 | 39.9 | <3.3 | 92 | 1.3 | <16.7 | <3.3 | 586 | 0.6 | 6.64 |
| 103 | <1.7 | <0.02 | <16.7 | 161 | <0.3 | <1.7 | 32.2 | <3.3 | 98 | 1.37 | <16.7 | <3.3 | 1020 | 1.0 | 8.31 |
| 104 | <1.7 | <0.02 | <16.7 | 102 | <0.3 | <1.7 | 49.8 | <3.3 | 100 | 1.74 | <16.7 | <3.3 | 750 | 0.8 | 10.2 |
| 105 | <1.7 | <0.02 | <16.7 | 158 | <0.3 | <1.7 | 26.7 | <3.3 | 1 | 35.8 | <16.7 | <3.3 | 3020 | 3.0 | 3.29 |
| 106 | <1.7 | <0.02 | <16.7 | 34.1 | <0.3 | <1.7 | 81 | <3.3 | 114 | 1.85 | <16.7 | <3.3 | 1520 | 1.5 | 10.6 |
| 107 | <1.7 | <0.02 | <16.7 | 242 | <0.3 | <1.7 | 22.4 | <3.3 | 109 | 1.54 | <16.7 | <3.3 | 636 | 0.6 | 7.43 |
| 108 | <1.7 | <0.02 | <16.7 | 166 | <0.3 | <1.7 | 38.8 | <3.3 | 4 | 0.423 | <16.7 | <3.3 | 1340 | 1.3 | 18.7 |
| 109 | <1.7 | <0.02 | <16.7 | 86 | <0.3 | <1.7 | 42.1 | <3.3 | 101 | 1.39 | <16.7 | <3.3 | 2260 | 2.3 | 6.3 |
| 110 | <1.7 | <0.02 | <16.7 | 147 | <0.3 | <1.7 | 32.2 | <3.3 | 109 | 1.41 | <16.7 | <3.3 | 3440 | 3.4 | 5.74 |
| 111 | <1.7 | <0.02 | <16.7 | 64.1 | <0.3 | <1.7 | BDL | <3.3 | 6 | 1.55 | <16.7 | <3.3 | 4310 | 4.3 | 5.26 |
| 112 | <1.7 | <0.02 | <16.7 | 47.1 | <0.3 | <1.7 | 46.5 | <3.3 | 96 | 2.45 | <16.7 | <3.3 | 3980 | 4.0 | 5.77 |
| 113 | <1.7 | <0.02 | <16.7 | 145 | <0.3 | <1.7 | 34.2 | <3.3 | 105 | 1.44 | <16.7 | <3.3 | 1340 | 1.3 | 6.96 |
| 114 | <1.7 | <0.02 | <16.7 | 99.8 | <0.3 | <1.7 | 54.7 | 18 | 152 | 2.67 | <16.7 | <3.3 | 772 | 0.8 | 8.13 |
| 115 | <1.7 | <0.02 | <16.7 | 74.6 | <0.3 | <1.7 | 57.7 | <3.3 | 138 | 1.89 | <16.7 | <3.3 | 1040 | 1.0 | 10.6 |
